# Breast abscess in two Nepalese newborns: An unusual series

**DOI:** 10.1016/j.amsu.2022.104774

**Published:** 2022-09-22

**Authors:** Ashish Lal Shrestha, Aakash Mishra

**Affiliations:** a Department of Pediatric and Neonatal Surgery, Kathmandu Medical College Teaching Hospital, Kathmandu, Nepal; b Kathmandu Medical College Teaching Hospital, Kathmandu, Nepal

**Keywords:** Neonate, Mastitis, Breast abscess, Suppurative mastitis, Case series

## Abstract

**Background:**

Breast abscess in newborns is an exceedingly rare pyogenic inflammation that usually starts as mastitis neonatorum. Although mastitis can respond to antibiotic therapy in the initial stages, once advanced into a purulent collection, decompression may be indicated either in the form of needle aspiration or even surgical drainage. We present two newborns with breast abscesses managed surgically with successful outcomes.

**Case presentation:**

Case 1: A 13-day-old boy presented with swelling and redness over the left breast for a day. Local examination revealed a warm, indurated swelling with redness and fluctuation. Needle aspiration confirmed pus. Intravenous (IV) antibiotics were started right away followed by pus drainage, the culture of which yielded Methicillin-Sensitive *Staphylococcus aureus* (MSSA). Regular wound care subsequently resulted in complete recovery.

Case 2: A 15-day-old boy presented with swelling, redness and milky discharge from the right breast for two days. The swelling was erythematous, tender and indurated but not cystic or fluctuant. Ultrasonogram was suggestive of an abscess. Once again, IV antibiotics were administered followed by drainage. Pus yielded MSSA. Following regular wound dressings, satisfactory healing was achieved.

**Conclusion:**

Neonatal mastitis and breast abscess are uncommon. Early recognition followed by appropriate antibiotic therapy and drainage of the abscess is the mainstay of treatment.

## Highlights

• Neonatal mastitis and breast abscess are fairly uncommon.
• Breast abscess, an exceedingly rare pyogenic inflammation, starts as mastitis neonatorum.
• Clinical improvement is aided by adequate antibiotic coverage and needle aspiration or surgical drainage of pus.
• Prompt recognition and management are paramount to avoid complications.
• Newborns with breast abscesses are managed surgically with successful outcomes.

## 1 Introduction

Breast enlargement of varying degrees in newborns, often called mastauxe, is not a rare event and is thought to result from *trans*-placentally acquired maternal estrogens. Contrary to this is neonatal suppurative mastitis, which is considered rather a rare phenomenon. The infection is mostly limited to the breast, although it can progress to cellulitis, fasciitis, osteomyelitis, brain abscess, and even generalized sepsis in rare situations [1,2]. We present two such cases of neonatal breast abscesses that were successfully treated with a combined approach. This work has been arranged in line with the PROCESS 2020 guidelines [3].

## 2 Case Presentation

### 2.1 Case 1

**Fig. 1:**
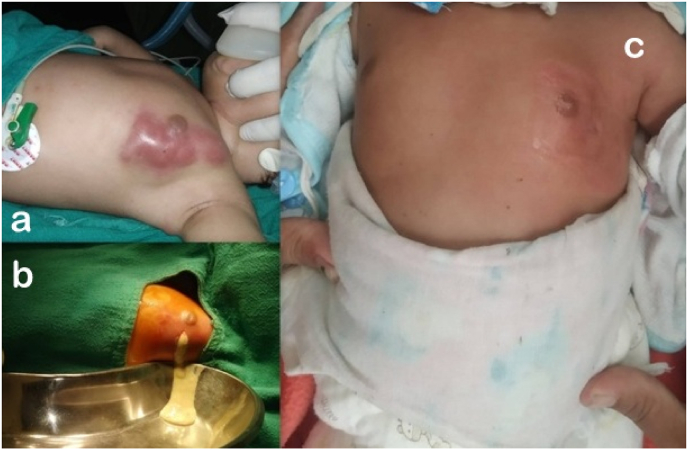
a: Left breast swelling with localized warmth and redness; b: Peroperative image showing 20ml of pus; c: Complete recovery at two weeks.

A 13-day-old boy born of normal delivery and uneventful antenatal history was brought in for swelling and redness over the left breast for a day. His mother admitted expressing the baby's breasts for ongoing milky nipple discharge. Examination revealed local warmth, redness and fluctuation over the swelling (Fig. 1a). The systemic examination was unremarkable.

He was started on intravenous (IV) Amoxycillin-Clavulanate. Diagnostic aspiration of the swelling confirmed pus. Incision and drainage of the left breast abscess revealed 20 ml of pus (Fig. 1b) in the subcutaneous plane, the culture of which yielded MSSA that was sensitive to first-line antibiotics.

Intravenous antibiotics were continued until cellulitis resolved following which he was discharged on oral antibiotics and advised to continue alternate day dressings with normal saline on as outpatient basis. The local findings subsided by two weeks of treatment (Fig. 1c).

### 2.2 Case 2

**Fig. 2:**
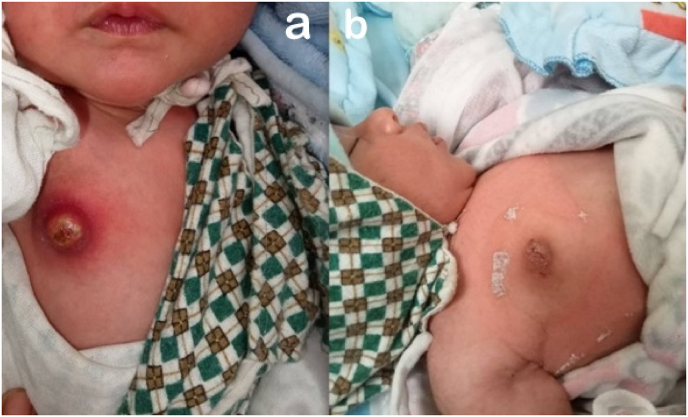
a: Right breast swelling with erythema, tenderness and induration; b: Complete recovery at two weeks.

A 15-day-old boy presented to the pediatric out-patient clinic with complaints of swelling and redness over the right breast for two days. His mother reported a milky discharge from the right breast. The prenatal history included normal pregnancy with no maternal infections and normal vaginal delivery. Local examination revealed swelling over the right breast with erythema, tenderness and induration (Fig. 2a). The systemic examination was unremarkable.

Ultrasonography (USG) of the right breast showed a well-defined heterogeneous lesion measuring 19 × 17 × 14 mm, predominantly hypoechoic with low-level internal echoes in the subcutaneous plane at the right nipple indicative of abscess formation. Again IV Amoxicillin-Clavulanate was initiated followed by incision and drainage of the right breast abscess. Around 15 ml of pus in the subcutaneous plane was obtained and sent for Culture and sensitivity. Postoperatively, IV antibiotic therapy was continued until cellulitis resolved. Following this, regular normal saline dressings were continued and he was discharged on oral antibiotics.

The culture again yielded Staphylococcus *aureus*, sensitive to Amoxicillin-Clavulanate. At two weeks, complete recovery was noted as shown in Fig. 2b.

## 3 Discussion

The evidence of breast inflammation, with or without abscess formation, within the first month of life, is called neonatal mastitis [1]. Neonatal mastitis occurs mostly in full-term infants, with a peak incidence at three weeks of age and is twice as common in female newborns [1,4]**.** *Staphylococcus aureus* is the most common etiologic agent and less commonly group B *Streptococcus*, group A *Streptococcus*, *Escherichia coli*, Bacteroides species, *Klebsiella pneumoniae*, and *Enterococcus* [1,4]. *Staphylococcus aureus* was cultured in both cases in our study. The potentially pathogenic bacteria colonizing the skin over the breast and nipple can travel in a retrograde fashion into the mammary glands and deeper tissues. Manipulation of the mammary gland as in, an attempt to express milk is a known risk factor for the development of purulent mastitis [1]. Hematogenous spread of infection is usually an exception.

The clinical presentation includes swelling, erythema, a local rise in temperature and induration. Although it can also be accompanied by changes in the surrounding skin and the involvement of the axillary lymph nodes, systemic signs of infection are uncommon [5]. It is mostly unilateral, with no right or left breast predilection. Newborn mastitis is often self-limiting and has a tendency to evolve into an abscess rarely. Both our newborns seemed to have undergone breast manipulation and subsequently developed an abscess.

The diagnosis of breast abscess is mostly clinical and when in doubt, USG findings of a collection can guide the appropriate line of treatment. Hematological workup to assess the severity of infection and microbiological study of pus in terms of culture and sensitivity can aid to the institution of suitable antibiotic therapy. USG evaluation seems to assist in two ways, firstly by identifying pus and secondly by confirming physiological breast hypertrophy or mastauxe [2].

In view of the chances of rapidly spreading infection, antibiotics with broader coverage are generally used aggressively that may have to be de-escalated once culture and sensitivity reports are ready. But the possibility of intervening in terms of needle aspiration or drainage is always kept in mind [5]. Empirical antibiotic therapy is targeted against common pathogens like Staphylococcus, as in our series, Amoxicillin-Clavulanate was started for both cases. Since the culture reports confirmed MSSA sensitive to the same, it could be continued in the oral form once cellulitis subsided.

## 4 Conclusion

Neonatal mastitis with breast abscess in a newborn is an uncommon condition. Prompt recognition and suitable management are paramount to avoid complications and morbidities.

## Provenance and peer review

Not commissioned, externally peer reviewed.

## Consent for publication

Written informed consent was obtained from the patient's guardians for publication of this case report and accompanying images. A copy of the written consent is available for review by the Editor-in-Chief of this journal on request.

## Ethical approval

This case series is exempt from ethical approval.

## Sources of funding

None.

## Author contribution

ALS conceived the study, was involved in patient care, supervised and provided valuable intellectual insights. AM drafted the manuscript.

## Registration of research studies

1. Name of the registry:
2. Unique Identifying number or registration ID:
3. Hyperlink to your specific registration (must be publicly accessible and will be checked):

## Guarantor

Dr. Ashish Lal Shrestha.

Department of Pediatric and Neonatal Surgery, Kathmandu Medical College Teaching Hospital,

Sinamangal, Kathmandu, PO Box- 12127, Nepal. Tel: +977-1-552-2981,

E-mail: ashishlalshrestha75@gmail.com.

## Consent

Written informed consent was obtained from the patient's guardians for publication of this case report and accompanying images. A copy of the written consent is available for review by the Editor-in-Chief of this journal on request.

## Declaration of competing interest

The authors declare that they have no known competing financial interests or personal relationships that could have appeared to influence the work reported in this paper.
